# Effect of Message Approach and Image Size on Pictorial Health Warning Effectiveness on Cigarette Pack in Indonesia: A Mixed Factorial Experiment

**DOI:** 10.3390/ijerph18136854

**Published:** 2021-06-25

**Authors:** Reny Yuliati, Billy Koernianti Sarwono, Abdillah Ahsan, I Gusti Lanang Agung Kharisma Wibhisono, Dian Kusuma

**Affiliations:** 1 Department of Communication, Faculty of Social and Political Science, Universitas Indonesia, Depok 16424, Indonesia; reny.yuliati87@ui.ac.id (R.Y.); billy.sarwono09@ui.ac.id (B.K.S.); i.gusti913@ui.ac.id (I.G.L.A.K.W.); 2 Faculty of Economics and Business, Universitas Indonesia, Depok 16424, Indonesia; ahsanov@yahoo.com; 3 Centre for Health Economics & Policy Innovation, Imperial College Business School, London SW7 2AZ, UK

**Keywords:** tobacco control, pictorial health warning, message approach, size, Indonesia

## Abstract

Background: Cigarette consumption remains high and increasing in Indonesia. The government implemented a pictorial health warnings requirement of 40% cover of the pack (front and back) using fear appeal messages. Objective: Our study aims to assess the effectiveness of cigarette pictorial health warnings by message and size. Methods: We conducted a mixed factorial experiment online study using three messaging approaches (fear vs. guilt vs. financial loss) and two picture sizes (40% vs. 75%) among 209 smoking participants. Sociodemographic variables included gender, education, income, employment status, and marital status. Data analysis used a mixed model ANOVA to see the main effect and interaction effect on dependent variables. For subgroup analysis, we used t-test and one-way ANOVA. All analyzes were in SPSS 22. Results: We found significant differences in the three message types, in which fear and guilt have higher effectiveness than financial loss. By subgroup, the guilt message was more compelling among female smokers and married smokers. The financial loss message was effective among lower-income smokers. We found no difference in pictorial health warning effectiveness by image size, potentially because participants could zoom in/out the cigarette pack image on the screen. Conclusions: Our finding supports more diverse message types in pictorial health warnings in Indonesia and other countries.

## 1. Background

Deaths due to tobacco consumption worldwide reach eight million people per year [1]. Despite the high mortality rate, the number of smokers remains high, including in Indonesia. In 2013, over 65 million adults smoked in Indonesia, ranked first in the Southeast Asian region [2]. The latest nationally representative data showed smoking prevalence was 61.4% and 2.3% among men and women aged 15+ years, respectively, in 2018 [3]. Data also showed the total spending on cigarettes in the country was more than that on food needed for nutrition [4]. Additionally, monthly expenditure per capita for cigarette and tobacco consumption reached 11.7%, while that for food such as cereals was 10.95%, and the meat was 4.7% [5].

The government has made efforts to reduce the consumption of tobacco products, including Pictorial Health Warning (PHW) on cigarette packs. PHW became mandatory per Health Law 36/2009 and Ministry of Health Regulation 28/2013 to include health warnings and information on tobacco product packaging. The actual warnings currently in use were updated by Ministry of Health Regulation 56/2017 and use a fear appeal approach. Current warnings include images of a man with large wound dressing on the chest and neck, and of a hole on a man’s neck. Meanwhile, the size of the PHW is relatively small (40% front and back) compared to other countries—Indonesia ranked 116th in the overall size in the world [6].

Recent studies have shown that PHWs are generally more effective than a textual warning. PHWs produce more negative effects in comparison to textual warnings [7,8]. PHWs were shown to have no impact on risk beliefs, such as the perceived likelihood of harm, perceived severity, and experiential risk [8]. However, in one study, negative effects could predict intention and smoking susceptibility, and indirectly predict these constructs through risk beliefs [9].

Previous studies have examined the content and format/size of persuasive messages. In terms of content, while the fear appeal is often used for anti-smoking advertisements [10], other emotional approaches include guilt and financial loss appeal. Guilt is characterized by a feeling that one has done or is doing something wrong and/or has engaged in a behavior that is immoral and harmful to others [11]. PHWs using guilt images are shown to be effective in causing guilt in young smokers and influencing their judgment in smoking. In the context of anti-smoking campaigns, messages that portray another life (e.g., a baby or fetus) might invoke a feeling of relevance and guilt [11,12]. Moreover, financial loss messages are important because smoking is detrimental to health and has implications in financial matters [13]. Studies suggest that a decrease in cigarette affordability results in decreased cigarette consumptions [14,15]. Therefore, financial concerns might be one type of message that could affect smokers, especially in Indonesia, where monthly expenditure per capita for cigarette and tobacco consumption reached 11.7% [5].

In terms of size, previous studies have shown mixed results. Bansal-Travers et al. [16] and Gravely et al. [17] showed that PHW size impacted smoking behavior by reducing cigarette consumption. Skurka et al. [18] also found that a larger size of PHW might increase the intention to quit smoking. However, Lacoste-Badie et al. [19] suggested that image size on cigarette packs has a low impact on smokers. Moreover, sociodemographic characteristics are also important, as messages might affect people differently according to their gender or age group. For example, Toerien et al. [20] found that some fear appeal frames, such as statement-based fear appeal, were more effective on females rather than males. Additionally, youth smokers in Singapore considered the fear appeal message irrelevant [12].

Those studies, however, have several limitations. First, they are mostly conducted in high-income countries. Although they were able to shed light on the role of PHW on several outcomes such as belief, affect, and behavior, studies from low- and middle-income countries (LMICs) are lacking. Secondly, they have not explored the effectiveness of financial loss PHW as an alternative on cigarette packs. Thus, our study aims to investigate the separate and combined effect of three types of message types (fear, guilt, and financial loss) and size (small and large) on the effectiveness of PHW. It also seeks to understand whether the different types of message appeals would affect sociodemographic characteristics differently.

## 2. Method

### 2.1. Study Design and Procedure

We used a mixed factorial experimental design of three message approaches (fear vs. guilt vs. financial loss) and two image sizes (small 40% vs. large 75% cover). The message approach was manipulated within-subject, while the image size was manipulated between-subject. In other words, all participants were shown the three message approaches, and each participant only saw one size.

To create stimuli that reflect cigarette pack warning images using fear, guilt, and financial approaches, we took the images from several sources. For images with fear approach, we adopted the PHW from other countries available on tobaccofreekids.org. For guilt and financial loss approaches, we obtained them from a royalty-free provider on shutterstock.com, which we edited to fit the context of PHW on cigarette packs. We did not use the images already used on cigarette packs in Indonesia to improve internal validity (i.e., many participants may have seen the Indonesian images). To select the images, we conducted a discussion with 30 postgraduate students at Universitas Indonesia. We provided three images for the students to choose from for each message approach (fear-, guilt-, and financial loss-appeal). After we explained the definition of each approach, the students discussed and rated each image (using a scale of 1–7). For each message approach, the image with the highest average rating was chosen. The chosen image for fear approached was rated 6.2, that for guilt approach 5.7, that for financial loss approach 6.3.

We included participants who were active smokers aged at least 18 years old. We used the G*Power analysis [21] for the variance analysis of 3 × 2 mixed factorial designs (α = 0.05, β = 0.9, ƒ = 0.2), suggested a minimum sample size of 178. In the data collection, we collected a total of 209 samples. Participants were recruited through a distribution of online participant recruitment posters with paid promotions on several social media (Instagram) accounts, including zonafotography, motretsuka.id, pulaumusik, musik_mellow, and musikstory. We chose Instagram to target youth and young adult active smokers. The Instagram accounts were chosen based on their popularity, marked by a large number of followers (over 100 thousand followers), and contents related to hobbies (photography and music, generally most relatable to young adults).

**Figure 1:**
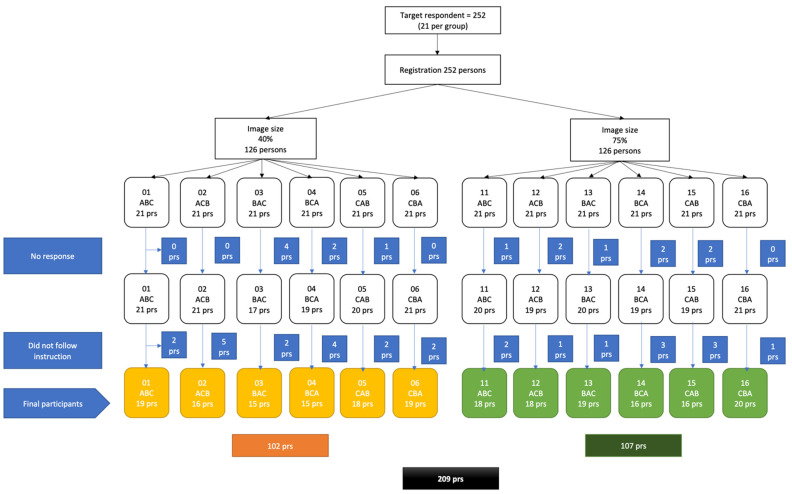
Assignment to condition and image counterbalancing. Note: A = Fear appeal image, B = Guilt appeal image, C = Financial loss appeal image. ABC, ACB, etc. show the order of images shown to each participant.

We provided a questionnaire link to those who agreed to participate in the study. The link led to one type of image size (40% or 75% cover) that was randomly allocated as participants registered themselves. Each participant was shown three message approaches: fear, guilt and financial loss by the counterbalanced treatment-order. Randomization was performed using the random.org website, which provides randomization tools such as coins and dice. Once participants clicked the link provided, they were asked to read a written consent form that briefly describes the study. On the consent form, participants were informed that the data collected online would be treated confidentially and there would be no penalty if participants decided to leave the study. After participants agreed to the consent form by clicking the button provided, they were asked to answer questions about their demographics and smoking habits. Then, on the next page, they were shown pictures of cigarette pack warnings (fear, guilt, and financial loss) alternately in counterbalanced order, followed by measures of the dependent variables. After participants completed the questionnaire, a debrief sheet appeared and participants were asked to type in their cellphone number to receive compensation for their time and effort (IDR 25,000 via GoPay online payment). Assignment to condition and image counterbalancing are provided in Figure 1.

### 2.2. Dependent and Independent Variables

The dependent variable is the effectiveness of PHW. After participants were exposed to cigarette pack images, they were asked to answer questions about the effectiveness of PHW on the cigarette pack. The measurement of PHW effectiveness was a modification of Kaplan et al. [22] by asking three questions: (1) makes me more worried when smoking, (2) makes me think twice about consuming cigarettes, and (3) motivates me to quit smoking. Each question was scaled from 1 (strongly disagree) to 7 (strongly agree). The three questions produced satisfactory results (mean = 4.36, SD = 1.15, and α = 0.80).

**Figure 2:**
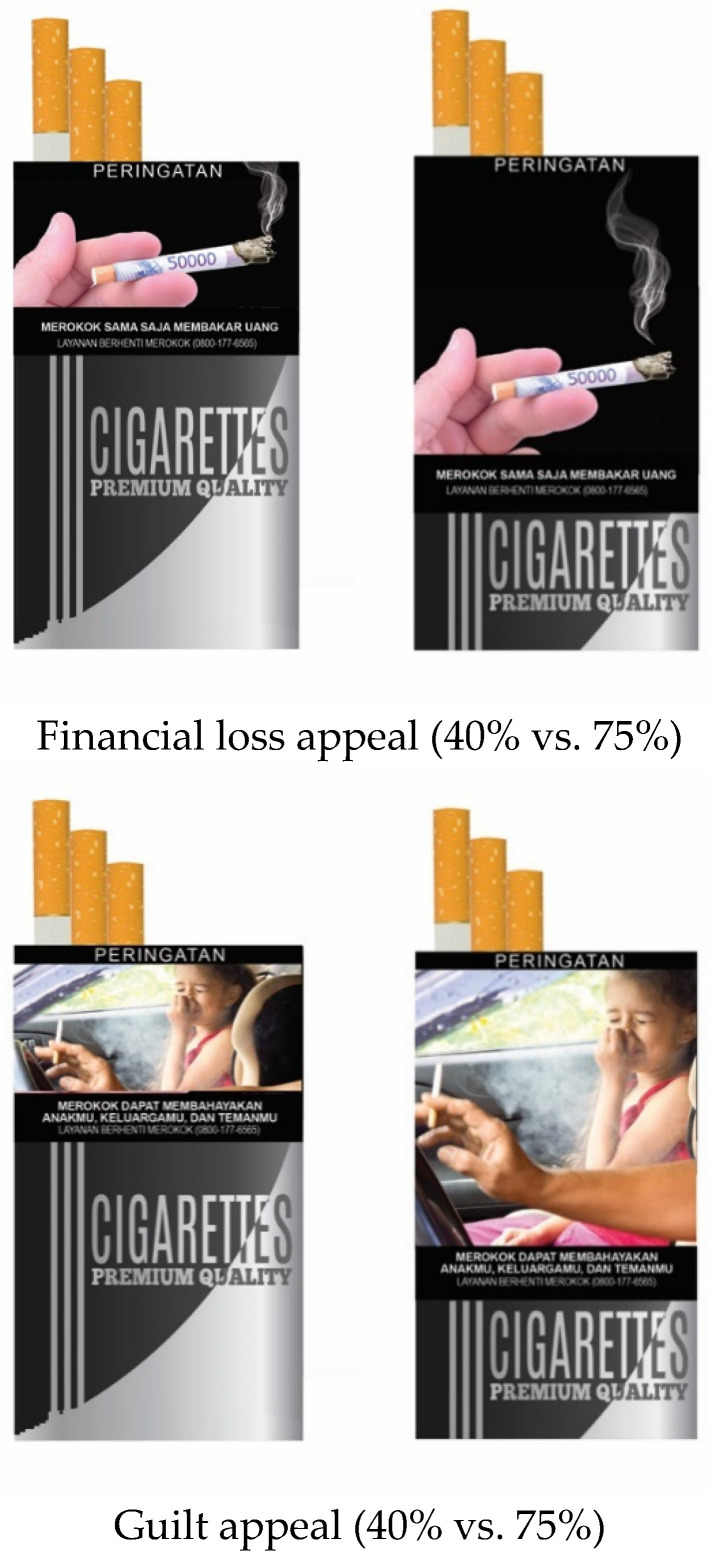

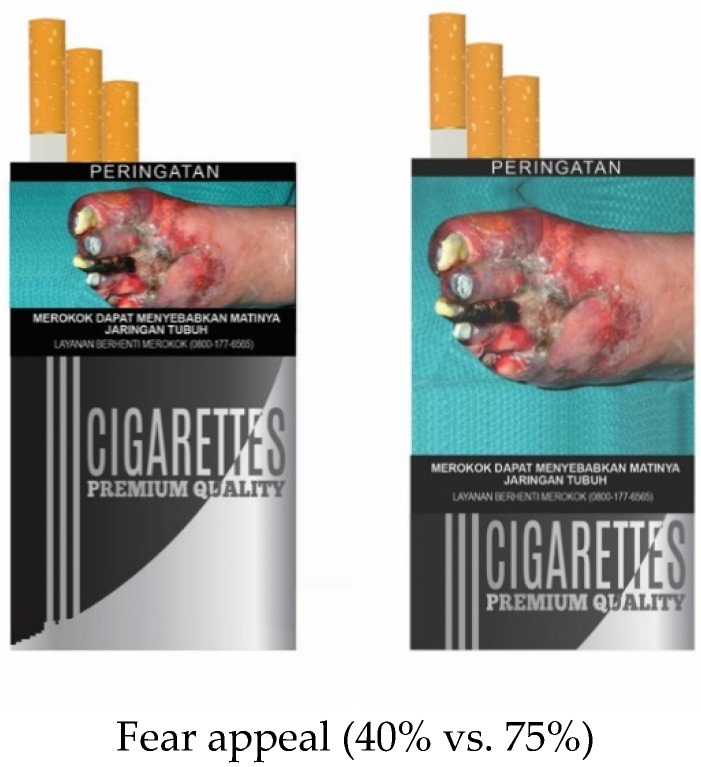
Experiment materials.

The main independent variables were message approaches and sizes. The message approach was manipulated within-subject. Participants were presented with a warning image on cigarette packs with three message approaches, namely messages of fear, guilt, and financial loss at the same time. The fear approach was displayed with a close-up of a body part suffering from gangrene, accompanied by text saying, “Smoking causes the death of body tissue.” For the guilty approach, a child was shown covering her nose because of cigarette smoke, accompanied by text saying, “Smoking can harm your child, your family, and your friends.” For the financial loss approach, an image of money in the forms of cigarette and burning was displayed, accompanied by text saying, “Smoking is burning money” (Figure 2).

Furthermore, the image size was manipulated between-subject. Participants were given a pictorial message warning with an image size of 40% or 75% of the cigarette pack cover. The 40% image size was chosen because it corresponds to the size of pictorial message warning on cigarette packs circulating in Indonesia, while 75% cover is the size of pictorial message warning that is enforced in various other foreign countries, namely: Canada, Myanmar, Brunei, Laos, and Tajikistan [6].

### 2.3. Data Analysis

We used a two-way mixed analysis of variance (ANOVA) to determine any main effects and interaction effects of the two independent variables toward dependent variables. To assess the differences by participant characteristics with each message approach, we used t-test and one-way ANOVA. All analyses used SPSS 22 (IBM Corp, Armonk, NY, USA) with a 5% level of statistical significance.

This study was approved by the Health Research Ethics Committee of the University of Prof. DR. Hamka (No: 03/20.11/0710).

## 3. Results

### 3.1. Descriptive Results

Table 1 shows the sample characteristics and PHW effectiveness. Panel (a) shows a total of 209 participants, including 87.6% were males and 12.4% females, with an average age of 25.9 (SD = 7.11) ranging from 18 to 63 years. More than half of the participants (58.9%) completed senior high school, 32.5% completed college, and 1.9% completed postgraduate. By income, 28.7% of participants earned under IDR 1 million, 34.0% of participants earned IDR 1 to 2.99 million, 24.9% participants earned IDR 3 to 4.99 million, and 12.4% participants earned more than IDR 5 million. The average number of cigarettes consumed per day was 11.53 (SD = 7.28). Moreover, Panel (b) provides the descriptive statistics (mean and standard deviation of the Likert scale) of PHW effectiveness as the dependent variables. PHW effectiveness was at a moderate level (mean = 4.36, SD = 1.15).

### 3.2. Main Analysis

The effectiveness of PHW score was analyzed by means of two-way mixed design ANOVA, with three levels of message types (fear, guilt, and financial loss) as a within-subjects factor and two levels of image size (40% and 75%) as a between-subjects factor. Mauchly’s test indicated that the assumption of sphericity had been violated, therefore degrees of freedom were corrected using the Greenhouse–Geisser estimates of sphericity.

Table 2 shows that there was a significant main effect of message approach (F (1.919, 414) = 10.371, *p* < 0.001, partial η^2^ = 0.048) on PHW effectiveness scores, with fear message (mean = 4.54, 95% CI: 4.32–4.76); guilt (mean = 4.52, 95% CI: 4.33–4.71) and financial loss (mean = 4.04, 95% CI: 3.82–4.26). A pairwise comparison showed that the fear and guilt messages were not significantly different (*p* = 0.875), but financial loss was significantly different from the other two messages (both *p* < 0.001).

In contrast, there was no significant main effect of image size on PHW effectiveness (F (1, 207) = 2.430, *p* = 0.121, partial η^2^ = 0.012), indicating a comparative PHW effectiveness of 40% (mean = 4.49, 95% CI: 4.26–4.71) and 75% (mean = 4.24, 95% CI: 4.02–4.45) were similar overall. There was also no significant interaction between message approach and image size (F (1.919, 414) = 1.79, *p* = 0.171, partial η^2^ = 0.009).

Table 3 shows t-test and one-way ANOVA by sample characteristics. To examine the differences by gender, we used student’s t-test; to examine the differences in other characteristics, we used a one-way ANOVA test for each characteristic in each message approach. For fear appeal, there were no specific characteristics to whom this message approach was more effective since all of the participants’ characteristics in this approach did not show any significant differences. For guilt appeal, results show it to be more effective among females (mean = 5.05, 95% CI: 4.48–5.62) than males (mean = 4.43, 95% CI: 4.23–4.63); *p* = 0.037. Guilt messages also seem to be effective among smokers who were married, especially with children, compared to those not married (*p* = 0.018). For the financial loss approach, a significant difference was found in income. Financial appeal tended to be more effective among those with a monthly income of IDR 1–5 million, but less so among those earned below IDR 1 million and over 5 million (*p* = 0.021).

## 4. Discussion

In this study, we aim to explore three message approaches and two image sizes in terms of PHW effectiveness using self-report measurement among adult active smokers. We found significant differences in the three message approaches, in which fear and guilt have higher effectiveness than financial loss. Additionally, the guilt message was more compelling among female smokers and married smokers, especially with children. The financial loss message was effective among low-income smokers. We found no difference in PHW effectiveness by image sizes, potentially because participants could zoom in/out the cigarette pack image on the screen. The following section discusses our notable findings.

First, the guilt message is more effective among females. This is in line with Brunel and Nelson’s study [23] that suggests that gender differences affect an individual’s response to the attractiveness of egotistical or altruistic messages, where women will tend to prefer a message of “helping others”, while men of “helping themselves.” A guilt message approach is a form of altruistic message, which is helping others rather than oneself. This is due to women being constructed to care and always relate to the views of society, unlike men [24]. Additionally, guilt message is more effective among families with children (compared to those who are single). This makes sense because the guilt approach on cigarette packs is usually associated with the negative effects of smoking that affect children’s health, inducing smokers’ guilty feelings. This aligns with previous studies showing that having children was one motivation to quit smoking among parents that smoke [25,26,27].

Second, although PHW effectiveness of financial loss messages is lower than that of fear and guilt approaches, the financial loss message is more effective when exposed to smokers with lower income (less than five million/month) than those with high income (above five million/month). Potentially, higher-income smokers are less burdened by the cigarette costs compared to lower-income smokers. Our findings also showed those with income less than one million/month have lower PHW effectiveness when exposed to the financial loss message. They are likely those without permanent jobs such as students that buy cigarettes using pocket money from parents. In our sample, the average income of students was IDR 705,000 (compared to IDR 3522,265 among employees). Moreover, previous studies showed that income is positively associated with youth smoking behavior in LMICs [28].

Third, we found that the fear message has no significant effect among any socioeconomic characteristics. In the literature, the effectiveness of the fear approach in inducing behavior change has generated a lot of debate. Our study, however, supports the notion that, in cigarette packs, the fear approach remains important and is generally effective across all demographics. This aligns with the arguments by Hastings and MacMadyen [29] that fear messages can be used like an alarm that goes off to notify the danger of smoking, and this alarm works for anyone regardless of particularities.

Fourth, we found that a larger PHW on cigarette packs has no significant difference in PHW effectiveness than smaller image size. Previous studies have mixed results. Lacoste-Badie et al. [19] suggested that image size on cigarette packs has a low impact on smokers, but Bansal-Travers et al. [16], Gravely et al. [17], and Skurka et al. [18] showed otherwise. For our findings, we conducted an online experiment study. This means that participants saw PHW messages on cigarette packs on a screen (not on the actual pack), which would allow them to zoom in/out the images. This might affect the insignificant results on image size. Other potential reasons for insignificant results include not using the actual cigarette pack [18] and a smaller sample size [19].

### 4.1. Study Limitations

Our study has at least four limitations. First, our study focused on active smokers aged 18 years and older. Future research should study young participants such as students that are vulnerable to becoming new smokers. Second, our study was conducted online so participants could zoom in on images, which may affect the results. Third, our study focused on PHW effectiveness measured by attitude (how PHW makes the individuals worry, thinks twice about consuming cigarette, and motivates to quit smoking). While some studies showed that intention and health behavior could be predicted by attitude, subjective norm, and perceived behavioral control [30,31,32], a recent literature review found that the link between these variables and anti-smoking interventions remains unclear [30]. Future research should examine specific health behavior such as smoking cessation or intention to quit smoking. Fourth, our study did not consider the severity level of pictorial warnings messages (i.e., each message approach may not have the same degree of severity). Future studies need to add the severity component.

### 4.2. Policy Implications

Our findings support policymakers to improve the diversity of PHW message approaches in Indonesia and other countries with similar settings. Additionally, our study provides evidence that specific PHW message approaches are more effective among certain demographics. Thus, more variety of scientifically proven PHW message approaches are needed for more effective tobacco control in Indonesia and beyond.

## 5. Conclusions

We found that PHW message approaches in fear, guilt, and financial loss themes differ in effectiveness, where fear and guilt have a higher effectiveness than the financial loss approach. The guilt message is effective among female smokers compared to male, those who were married. Meanwhile, the financial loss message is not effective among smokers with higher income. Furthermore, image size has no significant difference in the effectiveness of PHW messages in smokers, which might be attributed to the fact that we were not using an actual cigarette pack.

## Figures and Tables

**Table 1:** Characteristics of participants (all smoker) and PHW effectiveness.

**(a) Characteristics**		***n***	**%**
Gender	Male	183	87.6
	Female	26	12.4
Age	18–24 years	110	52.6
	25–30 years	62	29.7
	31–40 years	27	12.9
	≥41 years	10	4.8
Employment Status	Employed	120	57.4
	Student	68	32.5
	Unemployed	20	9.6
Monthly income	Less than 1 million	60	28.7
	1–3 million	71	34
	3–5 million	52	24.9
	≥5 million	26	12.4
Marital Status	Not yet married	147	70.3
	Married with no children	15	7.2
	Married with children	44	21.1
	Divorce/Widower/Widow	3	1.4
Last Education	Elementary School	3	1.4
	Junior High School	11	5.3
	High School	123	58.9
	Diploma/Bachelor	68	32.5
	Postgraduate	4	1.9
Smoking Status	Light (1–4 cigarettes/day)	31	14.8
	Medium (5–14 cigarettes /day)	120	57.4
	Heavy (>14 cigarettes/day)	58	27.8
**(b) Dependent Variable**		**Mean**	**SD**
PHW effectiveness	Total Effectiveness of PHW	4.36	1.15

Note: Monthly income was in Indonesian Rupiah (IDR). PHW = Pictorial Health Warning; SD = Standard deviation. Mean = average of Likert scores 1–7 (1 = strongly disagree, to 7 = strongly agree).

**Table 2:** Effectiveness of pictorial health warning by message approaches and image size.

Source	Sum of Squares	df		F Test	*p*-Value		Partial Eta Squared	
Main effect								
Message approaches	33.358	1.919		10.371	<0.001		0.048	
Image size	9.642	1		2.430	0.121		0.012	
Two-way interactions								
Message approaches by image size	5.743	1.919		1.785	0.171		0.009	
	Size 40%			Size 75%			Total mean of message approaches	
	mean	SE	95% CI	mean	SE	95% CI	mean	95% CI
Fear	4.79	0.16	(4.48–5.10)	4.28	0.15	(3.99–4.58)	4.54	(4.32–4.76)
Guilt	4.56	0.14	(4.29–4.83)	4.47	0.14	(4.20–4.74)	4.52	(4.33–4.71)
Financial loss	4.11	0.16	(3.80–4.42)	3.97	0.16	(3.66–4.28)	4.04	(3.82–4.26)
Total mean of image size	4.49	0.12	(4.26–4.71)	4.24	0.11	(4.02–4.45)		

Note: SE = Standard errors, df = degrees of freedom, CI = Confidence Interval

**Table 3:** Student’s T-test and one-way ANOVA among participant characteristics in the message approach.

Participants’ Characteristic	Fear	Results	Guilt	Results	Financial	Results
Sex						
Male	4.50 (1.61)	n.s.	4.43 (1.38)	t (207) = 2.10, *p* = 0.037 *	3.98 (1.63)	n.s.
Female	4.77 (1.68)		5.05 (1.49)		4.40 (1.60)	
Age						
18–24 years	4.52 (1.58)	n.s.	4.34 (1.40)	n.s.	4.00 (1.59)	n.s.
25–30 years	4.73 (1.35)		4.65 (1.37)		4.15 (1.50)	
31–40 years	4.35 (2.03)		4.84 (1.58)		4.15 (2.10)	
Beyond 40 years	3.90 (2.26)		4.73 (1.04)		3.43 (1.38)	
Income (per month)						
Less than 1 million	4.64 (1.44)	n.s.	4.19 (1.19)	n.s.	3.86 (1.43)	F (3205) = 3.30, *p* = 0.021 *
1–3 million	4.33 (1.76)		4.44 (1.46)		4.06 (1.70)	
3–5 million	4.75 (1.57)		4.87 (1.60)		4.53 (1.75)	
≥5 million	4.37 (1.68)		4.77 (1.11)		3.38 (1.11)	
Marriage Status						
Not yet married	4.45 (1.57)	n.s.	4.33 (1.36)	F(3205) = 3.43, *p* = 0.018 *	3.97 (1.58)	n.s.
Married with no children	4.96 (1.60)		4.87 (1.71)		3.71 (1.59)	
Married with children	4.64 (1.80)		4.92 (1.31)		4.30 (1.76)	
Divorcee/Widower/ Widow	4.56 (1.39)		5.89 (1.39)		5.11 (1.84)	
Last Education						
Elementary School	2.89 (1.71)	n.s.	3.67 (2.19)	n.s.	1.56 (0.51)	n.s
Junior High School	4.09 (1.90)		4.33 (1.61)		3.91 (1, 85)	
High School	4.60 (1.51)		4.52 (1.35)		4.07 (1.57)	
Diploma/Bachelor	4.52 (1.75)		4.57 (1.49)		4.16 (1.64)	
Postgraduate	4.92 (1.40)		4.58 (0.74)		3.25 (2.10)	
Smoking Status						
Light	4.81 (1.69)	n.s.	4.62 (1.42)	n.s.	4.24 (1.39)	n.s.
Medium	4.53 (1.61)		4.47 (1.42)		4.04 (1.71)	
Heavy	4.37 (1.59)		4.55 (1.39)		3.93 (1.58)	

Notes: n.s. is not significant. Income was in Indonesian Rupiah (IDR). * is significant at *p* < 0.05.

## Data Availability

Available upon reasonable request.
